# Multimodal Magnetic Resonance Imaging to Diagnose Knee Osteoarthritis under Artificial Intelligence

**DOI:** 10.1155/2022/6488889

**Published:** 2022-06-23

**Authors:** Zhiyan Zheng, Ruixuan He, Cuijun Lin, Chunyu Huang

**Affiliations:** ^1^Department of Radiology, Huizhou Municipal Central Hospital, Huizhou 516001, Guangdong, China; ^2^Department of Orthopaedic Surgery, Huizhou Municipal Central Hospital, Huizhou 516001, Guangdong, China

## Abstract

This work aimed to investigate the application value of the multimodal magnetic resonance imaging (MRI) algorithm based on the low-rank decomposition denoising (LRDD) in the diagnosis of knee osteoarthritis (KOA), so as to offer a better examination method in the clinic. Seventy-eight patients with KOA were selected as the research objects, and they all underwent T1-weighted imaging (T1WI), T2-weighted imaging (T2WI), fat suppression T2WI (SE-T2WI), and fat saturation T2WI (FS-T2WI). All obtained images were processed by using the I-LRDD algorithm. According to the degree of articular cartilage lesions under arthroscopy, the patients were divided into a group I, a group II, a group III, and a group IV. The sensitivity, specificity, accuracy, and consistency of KOA diagnosis of T1WI, T2WI, SE-T2WI, and FS-T2WI were analyzed by referring to the results of arthroscopy. The results showed that the peak signal-to-noise ratio (PSNR) and structural similarity index (SSIM) of the I-LRDD algorithm used in this work were higher than those of image block priori denoising (IBPD) and LRDD, and the time consumption was lower than that of IBDP and LRDD (*p* < 0.05). The sensitivity, specificity, accuracy, and consistency (Kappa value) of multimodal MRI in the diagnosis of KOA were 88.61%, 85.3%, 87.37%, and 0.73%, respectively, which were higher than those of T1WI, T2WI, SE-T2WI, and FS-T2WI. The sensitivity, specificity, accuracy, and consistency of multimodal MRI in diagnosing lesions in group IV were 95%, 96.10%, 95.88%, and 0.70%, respectively, which were much higher than those in groups I, II, and III (*p* < 0.05). In conclusion, the LRDD algorithm shows a good image processing efficacy, and the multimodal MRI showed a good diagnosis effect on KOA, which was worthy of promotion clinically.

## 1. Introduction

Knee osteoarthritis (KOA), also referred to as knee arthritis, is a common degenerative non-inflammatory disease in the clinic. It is mainly caused by the degeneration of cartilage in the knee joint, and it may involve the meniscus and bony ligament [1]. Nowadays, the aging of various diseases is becoming more and more serious, such as cardiovascular diseases [2], and KOA is also one of them because its incidence and damage will increase with age [3]. Therefore, KOA is more frequent in the elderly population [3]. However, with people's attention to fitness exercises in recent years, KOA caused by excessive joint load has trended to the young [4]. In the early stage of KOA, the water content of the articular cartilage is reduced, and the cartilage becomes thinner; in the later stage, cartilage disappears, and joints are narrowed, resulting in loss of joint function, which gives the patient a great quality of life. Therefore, timely diagnosis and treatment are necessary [5, 6].

The composition of cartilage is quite special. At present, magnetic resonance imaging (MRI) is the only imaging technology that can clearly display cartilage tissue in clinical examination. A large number of studies have shown that MRI has a good application effect in KOA, but there are differences in the diagnostic efficacy of images under different examination sequences, especially the high overlap in the image performance of conventional sequences [7–9]. In order to improve the diagnostic effect of MRI, the multimodal MRI technology is clinically proposed, which combines multiple imaging sequences and comprehensively analyzes its qualitative and quantitative parameters, and it is the best MRI diagnostic efficiency for diseases [10]. Clinically, according to the imaging principle of MRI, it is divided into three modalities: structure, diffusion, and perfusion. The conventional structure imaging sequence includes T1-weighted imaging (T1WI), T2-weighted imaging (T2WI), and dynamic enhanced magnetic resonance imaging (DCE-MRI); diffusion imaging sequences include diffusion-weighted imaging (DWI) and diffusion tensor imaging (DTI); and perfusion imaging sequences refers to the three-dimensional arterial spin-labeled perfusion imaging (3D-ASL) [11]. However, MRI images will be contaminated by noise during the imaging or transmission process, resulting in impaired image display quality. Therefore, research on denoising algorithms becomes of certain significance and value in order to obtain high-quality MRI.

With the rapid development of science and technology, artificial intelligence technology under deep learning has been widely used in imaging, providing solutions for the imbalance between the number of imaging doctors and clinical imaging data, the level of imaging doctors, and the allocation of resources. Deep learning methods have been fully and reasonably used in image processing and analysis in the medical field. In recent years, many denoising algorithms have been proposed, such as image block priori denoising (IBPD) algorithm [12] and low-rank decomposition denoising (LRDD) algorithm [13]. IBPD algorithm is often used in combination with the Gaussian mixture model (GMM) [14]. In research, GMM is often used to learn the priors of external noise-free image blocks and used for image denoising processing. The application research of the LRDD algorithm in image denoising processing is relatively extensive, but its application effect is limited [15,16]. Then, someone proposed an LRDD algorithm based on a noise-free image block prior algorithm (here, it is referred to as the I-LRDD algorithm). It was found that the denoising method could remove the noise and better retain the texture detail information of the image itself after experimental research [17].

In this work, 78 patients with KOA were selected as the research objects; multimodal MRI images based on I-LRDD were used to diagnose cartilage damage in KOA patients; and the diagnostic effects of single sequence MRI and multimodal MRI technology after algorithm processing were compared. It was hoped to improve people's understanding and application of imaging examination methods for KOA cartilage injury, especially multimodal MRI, and provide more effective diagnosis methods for patients.

## 2. Research Methods

### 2.1. Research Objects

In this work, 78 patients who were admitted to the joint surgery department of our hospital from March 2020 to September 2021 and diagnosed with KOA and underwent periarticular cartilage examination using arthroscopy were randomly selected as the objects. Among them, 44 were male patients and 34 were female patients, ranging in age from 32 to 70 years old, with an average age of 49.4 ± 15.17 years old. Cartilage was examined arthroscopically in 78 patients, including 198 cartilages in total (some patients examined multiple knee cartilage), of which 123 had lesions and 75 did not. All patients were scanned with conventional T1WI, T2WI, fat suppression T2WI (SE-T2WI) sequence, and fat saturation T2WI (FS-T2WI). The results of T1WI, T2WI, SE-T2WI, and FS-T2WI scans were set as T1WI group, T2WI group, SE-T2WI group, and FS-T2WI group, respectively. In addition, the result of comprehensive sequence diagnosis was set as a multimodal group. The T1WI, T2WI, SE-T2WI, and FS-T2WI single-sequence diagnosis results were compared with the comprehensive sequence diagnosis results, and the diagnosis effects of multimodal MRI under different articular cartilage lesions were evaluated by using the arthroscopic test results as the criteria. According to the grade of articular cartilage disease under arthroscope, the patients were rolled into groups I–IV. The diagnostic effect of multimodal MRI under different articular cartilage lesion grades was evaluated. This study had been approved by the relevant medical ethics committee.

Inclusion criteria were defined as follows: (1) all patients were over 18 years old; (2) all patients were diagnosed according to the *Guidelines for the Diagnosis and Treatment of Osteoarthritis in China (2019 Edition)* [18]; (3) all patients had complete MRI required; and (4) all patients had signed the informed consent forms.

Exclusion criteria were given as follows: (1) patients who could not undergo MRI examination; (2) patients with a history of high-intensity exercise training; and (3) patients with a clear history of trauma.

### 2.2. Imaging Examination

All patients were examined with the same instrument before and after treatment by an experienced (20 years or more of experience) technician in charge or deputy chief technician. The primary image interpretation and diagnosis were carried out by the attending physicians, and two senior doctors were invited to further interpret the results to guarantee the accuracy of the results. The examination instruments were Siemens MAGNETOM Prisma 3.0 T and PHILIPS Multira1.5 T. During the examination, the patient was instructed to take a supine position, and an 8-channel knee joint special coil was adopted for inspection. The scanning sequence was as follows: the conventional sagittal T1WI and T2WI, spin-echo T2WI (SE-T2WI) sequence of transverse, and coronal and oblique sagittal were performed firstly, and transect and coronal fat-saturated T2WI scans were then performed. The scanning parameters were set as follows: for the T1WI sequence: time of repetition (TR) was −14 ms, time of echo (TE) was −3.5 ms, and flip angle was −10° and 25°; for the T2WI sequence: TR was −42 ms, TE was −10 ms, 20 ms, 40 ms, and 55 ms; and for FS-T2WI sequence: field of view (FOV) was 20, layer thickness was 3 mm, layer spacing was 0.5 mm, TE was 85.0, and matrix was 288 × 192. MRI postprocessing workstation (Siemens MultiModality Workplace) was used for image reconstruction, and MRI low-rank decomposition denoising algorithm based on noised image block prior was used for image feature extraction and denoising. The IBPD and LRDD algorithms were used to process the image and compared with the algorithm adopted in this work.

### 2.3. Images' Preprocessing

The working process of the multimodal MRI-based LRDD algorithm was described as follows. The first step was to use the GMM model to learn the MRI IBPD. The second step was to use the GMM obtained in the first step to cluster the noisy MRI image blocks. After clustering, it should stack the MRI image blocks in the Gaussian class to form a low-rank matrix and use the low-rank decomposition method for denoising. The third step was to reconstruct a clear MRI image based on the denoised data.

**Step 1 .**

It was assumed that the original MRI image was *Q*, it would be overlapped and cut into *m* image blocks of the same size; then a set can be formed as follows:

(1) $$\begin{array}{c} RQ=\left(R_{1}Q,\text{…}R_{i}Q,\text{…},R_{m}Q\right), \end{array}$$

where *R*_*1*_*Q* represents the *i*th image block in the image *Q*. If the set *RQ* is divided into *K* classes and there are *K* Gaussian classes in GMM, then the probability of any image block *R*_*i*_*Q* in *RQ* can be expressed as follows:

(2) $$\begin{array}{c} p\left(R_{i}Q|\Theta \right)=\sum_{k=1}^{K}w_{k}p_{k}\left(R_{i}Q|\mu_{k},\sum k\right), \end{array}$$

where Θ=(*μ*_1_,…, *μ*_*k*_, ∑1,…, ∑*k*, *w*_1_,…, *w*_*k*_) represents the parameter set of GMM, *w*_*k*_ represents the weight of the *k*th Gaussian distribution, *μ*_*k*_ refers to the mean of the *k*th Gaussian distribution, ∑*k* represents the covariance matrix of the *k*th Gaussian distribution, and *p*_*k*_(*R*_*i*_*Q|μ*_*k*_, ∑*k*) represents the density function of the *k*-th Gaussian distribution. *p*_*k*_(*R*_*i*_*Q|μ*_*k*_, ∑*k*) could be expressed as follows:

(3) $$\begin{array}{c} P_{k}\left(R_{i}Q|\mu_{k},\sum k\right)=c\times \;\exp\left(-\frac{1}{2}{\left(R_{i}Q-\mu_{k}\right)}^{T}\sum_{K}^{-1}\left(R_{i}Q-\mu_{k}\right)\right), \end{array}$$

where *c* represents the normalization constant, and the negative exponent in (−1/2(*R*_*i*_*Q* − *μ*_*k*_)^*T*^∑_*K*_^−1^(*R*_*i*_*Q* − *μ*_*k*_)) represents the Mahalanobis distance between *R*_*i*_*Q* and *μ*_*k*_

In order to make the expression of the calculation formula simpler, the Gaussian class to which each image block belongs was expressed as *C*=(*c*_1_, *c*_2_,…, *c*_*m*_), *c*_*i*_ ∈ {1,2,…, *K*}; then, under the parameter set Θ of GMM, the probability of the *k*-th Gaussian class in *R*_*i*_*Q*(*i*=1,…, *m*) can be expressed as *p*(*R*_*i*_*Q*, *c*_*i*_=*k|*Θ). If *R*_*i*_*Q* and *R*_*j*_*Q*(*i*, *j*=1,…, *m*, *i* ≠ *j*) were independent of each other, then the probability that the MRI image block set RQ under Θ was clustered into *K* classes can be expressed as follows:

(4) $$\begin{array}{c} p\left(RQ,C|\Theta \right)=\prod_{i=1}^{m}p\left(R_{i}Q,c_{i}|\Theta \right). \end{array}$$

After the logarithmic conversion of formula (4), the following equation could be obtained:

(5) $$\begin{array}{c} \log\;p\left(RQ,C|\Theta \right)=\sum_{i=1}^{m}\log\;p\left(R_{i}Q,c_{i}|\Theta \right), \end{array}$$

and

(6) $$\begin{array}{c} \log\;p\left(RQ,C|\Theta \right)=\sum_{i=1}^{m}\log\;p\left(c_{i}\right)p\left(R_{i}Q|c_{i}\right). \end{array}$$

The following equation could be obtained by combining (2) with (6):

(7) ∑i=1mlog  pcipRiQ|ci=∑i=1mlog  wcipciRiQ|μ,ci∑ci.

**Step 2 .**

It was assumed that a noisy MRI image was *Y*, and the image block set obtained from its segmentation was *RY*=(*R*_1_*Y*,…, *R*_*i*_*Y*,…, *R*_*m*_*Y*). When the prior information of the noise-free MRI image learned by GMM was known, *RY* was divided into *K* categories; then the matrix composed of all image blocks in the *k*-th category can be expressed as ${\bar{R}}_{k}Y=\left(R_{k},\text{…},R_{k\left(d\right)k}\right)$, where *d*(*k*) represents the number of similar blocks in the *k*-th class. The image blocks in the same Gaussian class had similar information, so ${\bar{R}}_{k}Y$ can be expressed as follows:

(8) $$\begin{array}{c} {\bar{R}}_{k}Y=Z_{k}+N_{k}, \end{array}$$

where *Z*_*k*_ represents the low-rank matrix and *N*_*k*_ represents the noise matrix. The noise on each pixel in the MRI image was assumed to be independently distributed; then, the following equation could be acquired based on the conditional possibility:

(9) $$\begin{array}{c} p\left({\bar{R}}_{k}Y|Z_{k}\right)\propto \;\exp\left(-\frac{1}{\sigma^{2}}{\left\|{\bar{R}}_{k}Y-Z_{k}\right\|}_{F}^{2}\right). \end{array}$$

Then, it could minimize (9) to obtain the following equation and the value of *Z*_*k*_:

(10) $$\begin{array}{c} E\left(Z_{k}\right)=\tau {\left\|Z_{k}\right\|}_{*}+\frac{1}{\sigma^{2}}{\left\|{\bar{R}}_{k}Y-Z_{k}\right\|}_{F}^{2}, \end{array}$$

where *τ* represents the normal number, *σ* represents the noise standard deviation, ‖*·*‖_*∗*_ referred to the kernel norm of the matrix, and ‖.‖_*F*_ was the Frobenius norm of the matrix. According to the minimization problem shown in the above equation, the optimization solution was carried out through the weight kernel norm. *U*∑*V*^*T*^ was supposed to represent the SVD decomposition of ${\bar{R}}_{k}Y$; then the following equation could be given:

(11) $$\begin{array}{c} Z{\bar{\bar{R}}}_{k}=US_{w}\left(\sum V^{T}\right), \end{array}$$

where *S*_*w*_(∑) represents the singular value contraction operator.

Combining the above analysis, it can reconstruct noise-free MRI images according to the following objective function:

(12) $$\begin{array}{c} \left(\bar{\bar{X}},\bar{\bar{C}},\left\{{\bar{\bar{Z}}}_{k}\right\}\right)=\underset{X,C,\left\{Z_{k}\right\}}{\arg\min\frac{\lambda }{\sigma^{2}}}{\left\|Y-X\right\|}_{2}^{2}-\log\;p\left(RY,C|\Theta \right)+\sum_{k=1}^{K}E\left(Z_{k}\right), \end{array}$$

where *λ* and *σ* represents the normal number and the noise standard deviation, respectively.

**Step 3 .**

The MRI image was reconstructed according to the denoised data combined with the above algorithm.

To evaluate the effect achieved by the above algorithm models, the denoising performance was evaluated by the peak signal-to-noise ratio (PSNR), structural similarity (SSIM), and convergence time. The smaller the value of PSNR, the lower the degree of image distortion. The closer the value of SSIM is to 1, the more similar the processed image is to the original image. They were expressed as follows:

(13) $$\begin{array}{c} \text{PSNR}=10\;\log_{10}\left(\frac{M}{{\left\|Y-\bar{\bar{Y}}\right\|}_{2}^{2}}\right), \end{array}$$

where *Y* represents the noise-free MRI image, $\bar{\bar{Y}}$ represents the denoised image, and *M* represents the number of pixels in the image.

(14) $$\begin{array}{c} \text{SSIM}\left(x,y\right)=\frac{\left(2\alpha_{x}\alpha_{y}+e_{1}\right)\left(2\beta_{xy}+e_{2}\right)}{\left(\alpha_{x}^{2}+\alpha_{y}^{2}+e_{1}\right)\left(\beta_{x}^{2}+\beta_{y}^{2}+e_{2}\right)}, \end{array}$$

where *α*_*x*_ and *α*_*y*_ represent the average value of *x* and *y*, respectively; *β*_*x*_^2^ and *β*_*y*_^2^ refer to the variance of *x* and *y*, respectively; and *β*_*xy*_ represents the covariance of *x* and *y*.

### 2.4. Observation Indicators

The arthroscopic diagnosis was undertaken as a criterion to evaluate the imaging characteristics and diagnostic efficacy (sensitivity, specificity, accuracy, and Kappa value) of five MRI methods (T1WI, T2WI, SE-T2WI, FS-T2WI single sequence, and multimode sequence scan) and multimode MR in different grades of cartilage injury (group I, group II, group III, and group IV).

### 2.5. Statistical Methods

Statistical analysis was performed using SPSS 22.0 software. Different sequences of MRI diagnosed different grades of cartilage damage accuracy were compared using the *χ*^2^ test, and when *p* < 0.05, statistical differences were considered. Kappa test was used to evaluate the consistency of cartilage injury diagnosis and arthroscopic results comparison among different sequences of MRI examination. Kappa value < 0.45 was considered to be poor or general; 0.45∼0.75 was considered to be strong; and >0.75 was considered to be strong.

## 3. Results

### 3.1. Evaluation of the Effect of Computer Image Preprocessing

**Figure 1:**
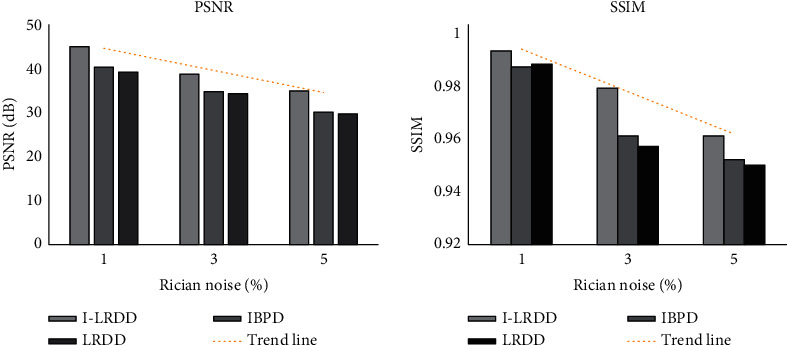
Comparison on denoising effects of computer image processing technology.

**Figure 2:**
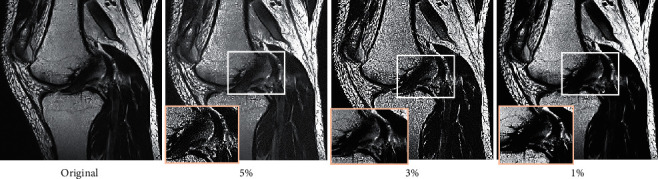
Image preprocessing effect based on computer technology: (a) original, (b) 5%, (c) 3%, and (d) 1%.

In order to evaluate the application effect of the I-LRDD algorithm, it was compared with image block prior denoising algorithm (IBPD) and low-rank decomposition denoising algorithm (LRDD) on real MRI data. As shown in Figure 1, when the rice intensity was 1%, 3%, and 5%, the PSNR values of the I-LRDD algorithm were 0.993, 0.979, and 0.961, respectively, which were significantly higher than those of IBPD and LRDD algorithms (*p* < 0.05). As illustrated in Figure 2, the lower the rice intensity was, the better the denoising effect was. When the rice intensity was 1%, the MRI image quality processed by the I-LRDD algorithm was closest to that of the noise-free image, and the processing effect was the best.

### 3.2. General Information of Patients

**Figure 3:**
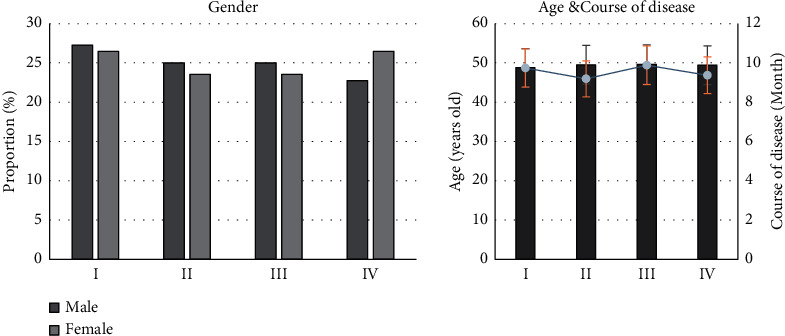
Distribution of general data of patients.

The distribution of patients in groups I, II, III, and IV was shown in Figure 3. There were 12 male patients in group I (27.27%), 11 cases in group II (25%), 11 cases in group III (25%), and 10 cases in group IV (22.73%). In addition, there were 9 female patients in group I (26.47%), 8 cases in group II (23.53%), 8 cases in group III (23.53%), and 9 cases in group IV (26.47%). The average age distribution showed 48.79 ± 15.45 years old in group I, 49.52 ± 15.12 years old in group II, 49.62 ± 14.35 years old in group III, and 49.42 ± 13.87 years old in group IV. The average course of disease showed 9.74 ± 1.17 months in group I, 9.19 ± 0.87 months in group II, 9.88 ± 0.98 months in group III, and 9.37 ± 1.32 months in group IV. After comparative analysis, it was found that there was no significant difference in gender, average age, and the average course of disease among different groups of patients (*p* > 0.05), as shown in Figure 3, indicating that the comparison was feasible.

### 3.3. Comparison of Diagnostic Effects

The diagnostic results of the T1WI sequence MRI scan and arthroscopy were shown in Table 1. After calculation, it can be concluded that the diagnostic sensitivity, specificity, accuracy, and consistency (Kappa value) were 47.15%, 46.67%, 46.97%, and 0.37, respectively.

The diagnostic results of the T2WI sequence MRI scan and arthroscopy were shown in Table 2. After calculation, it can be concluded that the diagnostic sensitivity, specificity, accuracy, and consistency (Kappa value) were 52.03%, 53.33%, 52.53%, and 0.41, respectively.

The diagnostic results of the SE-T2WI sequence MRI scan and arthroscopy were shown in Table 3. After calculation, the diagnosis sensitivity, specificity, accuracy, and consistency (Kappa value) were 56.91%, 57.33%, 57.07%, and 0.43, respectively.

The diagnostic results of FS-T2WI sequence MRI scan and arthroscopy were shown in Table 4. After calculation, the diagnosis sensitivity, specificity, accuracy, and consistency (Kappa value) were 61.79%, 60%, 61.11%, and 0.46, respectively.

The diagnostic results of multimodal MRI scan and arthroscopy were shown in Table 5. After calculation, it can be concluded that the diagnostic sensitivity, specificity, accuracy, and consistency (Kappa value) were 88.61%, 85.33%, 87.37%, and 0.73, respectively.

**Figure 4:**
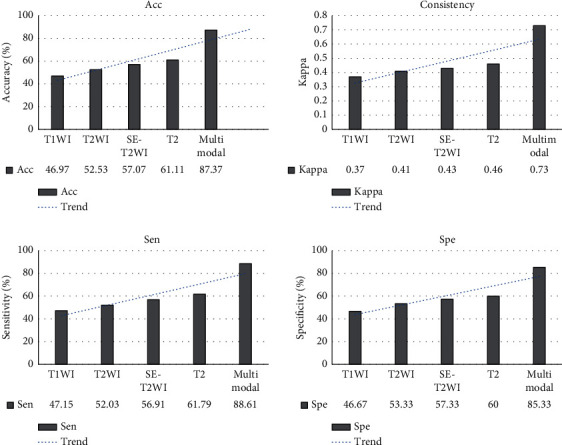
Comparison of MRI diagnostic effects of different sequences.

**Figure 5:**
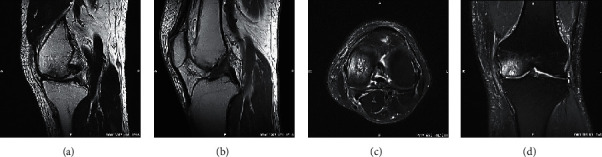
MRI scan images: (a) sagittal T1WI, (b) sagittal T2WI, (c) cross-sectional SE-T2WI, and (d) coronal SE-T2WI.

As illustrated in Figures 4 and 5(d), the comparison showed that in the T1WI group, T2WI group, SE-T2WI group, FS-T2WI group, and multimodal MRI group, the sensitivity, specificity, accuracy, and consistency of the five groups of inspection methods were in upward trends. However, there was no significant difference among the first four groups (*p* > 0.05), and those of the multimodal MRI group were significantly better than those in the T1WI group, T2WI group, SE-T2WI group, and T2 group (*p* < 0.05).  Figure 5 illustrated the KOA scan images of different sequences of MRI. The damage to articular cartilage can be observed in Figure 5; both T1WI and T2WI showed low signal, while the SE-T2WI sequence showed a high signal shadow.

### 3.4. The Diagnostic Effect of Multimodal MRI on Cartilage Lesions of Different Grades

**Figure 6:**
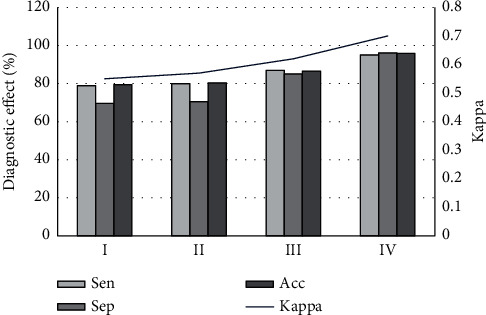
The diagnostic effect of different grades of cartilage lesions.

After arthroscopy, the results showed that among the 123 cartilage lesions, there were 36 articular cartilage lesions in grade I, 27 in grade II, 31 in grade III, and 29 in grade IV. Multimodal MRI and arthroscopy were used to analyze the diagnosis results of cartilage lesions of different grades, as shown in Figure 6. The results showed that the sensitivity, specificity, accuracy, and consistency of multimodal MRI for the diagnosis of group I lesions were 78.96%, 69.61%, 79.44%, and 0.55, respectively; the four indicators of group II lesion diagnosis were 80%, 70.53%, 80.35%, and 0.57, respectively; those in group III lesion diagnosis were 86.96%, 85.14%, 86.46%, and 0.68, respectively; and those of group IV lesion diagnosis were 95%, 96.10%, 95.88%, and 0.70, respectively. It suggested that the diagnosis rate of group IV was significantly higher than that of the other three groups, while that in group III was higher than the other two groups (*p* < 0.05), but there was no significant difference between groups I and II (*p* > 0.05).

## 4. Discussion

The occurrence of KOA is closely related to the degenerative damage of knee cartilage. MRI, as the only scanning method that can clearly show the cartilage lesions of the joint, has been extensively studied in clinical practice. However, conventional MRI examinations are often ignored due to thicker scans, small lesions, and failing to accurately diagnose early lesions of articular cartilage in patients with KOA [19]. Therefore, the multimodal MRI technology was proposed in this work, aiming to understand its application effect in the diagnosis of cartilage damage grade in KOA patients.

In order to make the results of the study more accurate, the MRI LRDD algorithm based on IBPD was adopted to process the MRI of KOA patients, and the denoising effects were analyzed and compared. The results showed that when the rice intensity was 1%, 3%, and 5%, the PSNR values (44.89 dB, 38.67 dB, and 34.88 dB), SSIM (0.993, 0.979, and 0.961), and consumption time (1.761 s, 1.981 s, and 2.121 s) of the algorithm adopted in this work were better than IBPD and LRDD under the corresponding rice intensity (*p* < 0.05), suggesting that the denoising effect of the two algorithms combined was better than that of a single method. Xie et al. (2020) [20] proposed that the effectiveness of the LRDD algorithm was still worthy of improvement. The IBPD method in this study was used for learning through GMM, which indirectly indicates the effectiveness of GMM. Many studies have shown that GMM is very good in the optimization effect of the algorithm [21], suggesting that the results of this study are precious. However, analysis of the research results revealed that with the increase of rice intensity, the PSNR value and SSIM result of the algorithm adopted in this work, IBPD algorithm, and LRDD algorithm gradually decreased, and the time consumption of the algorithms gradually increased. It shows that the application of the above algorithms is limited by the noise intensity. The higher the rice intensity, the worse the denoising effect and performance, which requires further optimization.

Comparison results of multimodal MRI technology with a single MRI scan sequence revealed that the diagnostic sensitivity, specificity, accuracy, and consistency (Kappa value) of the multimodal group were 88.61%, 85.33%, 87.37%, and 0.73, respectively. The results showed that the combination of multiple MRI sequences in the multimodal technique was more effective in the diagnosis of knee osteoarthritis and chondropathy. Ohno et al. [22] proposed in their study that MRI, FDG PET/MRI, and FDG PET/CT have higher diagnostic accuracy in TNM staging than conventional imaging examinations. In addition, Gui et al. [23] proposed in their study that a multimodal MRI scan has a diagnostic sensitivity of 68.8%, specificity of 72.5%, and accuracy of 70.5% for diseases, which were lower than the values in this work, suggesting that artificial intelligence algorithm has the role of improving MRI diagnosis effect. Borić et al. [24] also proposed multimodal MRI as an effective means of evaluating cartilage lesions, and the results were basically consistent with the results of this work. Moreover, multimodal MRI technology not only has a good application effect in the diagnosis of KOA cartilage lesions but also has good development prospects in the application of multimodal MRI technology in breast diseases [25], brain diseases [26,27], tumor diseases [28], and other aspects. The above results show that multimodal MRI has good application advantages in disease diagnosis. During the research process, multimodal MRI technology was also used to diagnose different grades of knee cartilage lesions, and the grading results under arthroscopy were used as the standard for evaluation. The results showed that the sensitivity, specificity, accuracy, and consistency of multimodal MRI in the diagnosis of group IV lesions were 95%, 96.10%, 95.88%, and 0.70, respectively, which were significantly higher than those of groups I, II, and III (*p* < 0.05). However, the sensitivity, specificity, and accuracy in group III (86.96%, 85.14%, and 86.46%) were higher than those of groups I and II, indicating that the higher the lesion grade, the better the diagnostic effect of multimodal MRI. However, the results of Wei et al. (2019) [29] showed that the change of the sensitivity of the knee cartilage by quantitative magnetic susceptibility mapping decreased with the increase of cartilage degeneration, which is contrary to the results of this work. Such inconsistent results may be caused by the difference between MRI and quantitative magnetic susceptibility mapping detection principle and the difference in sensitive substances to the human body. The research results of Spahn et al. [30] are consistent with the conclusions of this work, but because there are relatively few similar studies, this conclusion still needs further verification.

## 5. Conclusion

This work not only processed the diagnostic effect of MRI images of various sequences based on the I-LRDD denoising algorithm in cartilage injury of KOA patients but also analyzed the diagnostic effect of multimodal MRI in cartilage injury of different levels, so it was relatively more comprehensive. According to the research results, the LRDD algorithm based on noised image block prior showed a good image processing effect, and the diagnostic sensitivity, specificity, accuracy, and consistency (Kappa value) of multimodal MRI (88.61%, 85.3%, 87.37%, and 0.73%, respectively) were better than that of single scan sequence, which was more conducive to the diagnosis of KOA cartilage lesions. Therefore, it was worthy of clinical application and promotion. Moreover, the higher the cartilage lesion grade, the better the diagnostic effect of multimodal MRI. However, the number of patients in each group was too small, and the selection range of research objects was limited, leading to the lack of accuracy and representativeness of the results, which required further expansion. This work proved that the combined use of multiple MRI sequences for disease diagnosis was more conducive to the diagnosis and treatment of doctors, and its clinical development prospects were very promising.

## Figures and Tables

**Table 1:** The statistics of diagnosis results using T1WI sequence MRI scan and arthroscopic diagnosis results.

		Arthroscopic diagnosis results (*n* = 198 cases)		Total
		KOA	Non-KOA	
T1WI sequence (*n* = 198 cases)	KOA	58	40	98
	Non-KOA	65	35	100

Total		123	75	198

**Table 2:** The statistics of diagnosis results using T2WI sequence MRI scan and arthroscopic diagnosis results.

		Arthroscopic diagnosis results (*n* = 198 cases)		Total
		Non-KOA	Non-KOA	
T2WI sequence (*n* = 198 cases)	KOA	64	35	99
	Non-KOA	59	40	99

Total		123	75	198

**Table 3:** The statistics of diagnosis results using SE-T2WI sequence MRI scan and arthroscopic diagnosis results.

		Arthroscopic diagnosis results (*n* = 198 cases)		Total
		KOA	Non-KOA	
SE-T2WI sequence (*n* = 198 cases)	KOA	70	32	102
	Non-KOA	53	43	96

Total		123	75	198

**Table 4:** The statistics of diagnosis results using FS-T2WI sequence MRI scan and arthroscopic diagnosis results.

		Arthroscopic diagnosis results (*n* = 198 cases)		Total
		KOA	Non-KOA	
Fat-saturated T2WI (*n* = 198 cases)	KOA	76	30	106
	Non-KOA	47	45	92

Total		123	75	198

**Table 5:** The statistics of the diagnosis results of multimodal MRI scan and arthroscopic diagnosis.

		Arthroscopic diagnosis results (*n* = 198 cases)		Total
		KOA	Non-KOA	
Multimodal sequence (*n* = 198 cases)	KOA	109	11	110
	Non-KOA	14	64	88

Total		123	75	198

## Data Availability

The data used to support the findings of this study are included within the article.
